# The Efficacy of Biofeedback Therapy for the Treatment of Fecal Incontinence After Soave Procedure in Children for Hirschsprung's Disease

**DOI:** 10.3389/fped.2021.638120

**Published:** 2021-08-26

**Authors:** Yuhang Yuan, Mengyao Xu, Heying Yang, Beibei Sun, Yanan Li, Ning Zhang, Guantao Wang, Fan Su

**Affiliations:** Department of Pediatric Surgery, The First Affiliated Hospital of Zhengzhou University, Zhengzhou, China

**Keywords:** biofeedback therapy, Hirschsprung's disease, fecal incon tinence, individual treatment, child

## Abstract

**Introduction:** Hirschsprung's disease is a common digestive tract malformation in children, and the Soave procedure is one of the classic surgical methods for Hirschsprung's disease (HD). Fecal incontinence is one of the most common postoperative complications that can cause significant distress to the patients and their family, the incidence of which is 20% in a recent series. Biofeedback therapy (BFT) can be an effective treatment for managing anorectal disorders, but there has been little report of the efficacy of BFT for the treatment of fecal incontinence after the Soave procedure, and the main objective of this study is to evaluate it.

**Methods:** We retrospectively analyzed postoperative fecal incontinence in 46 children who received the Soave procedure for HD and who received BFT at our institution from March 2016 to February 2020, which included 38 males and 8 females (mean age 8.1 years, from 3.7 to 14 years). Anal sphincter contraction training was performed using BFT for 10 days per session in the hospital, one time each day, and 20 min each time. BFT was performed by employing visual and verbal feedback techniques using the biofeedback instrument. Long-term functional outcomes were objectively assessed using the Rintala Bowel Function Score (RBFS), and the patients were scored according to the sum total as excellent (18–20 points, 0 case), good (11–16 points, 0 case), fair (9–11 points, 9 cases), or poor (6–9 points, 37 cases). Defecation questionnaires and anorectal manometry were completed pretreatment and after three, six, or nine sessions, and primary outcome measures of anorectal manometry were anal maximal contraction pressure (AMCP), anal longest contraction time (ALCT), rectal rest pressure (RRP), and anal rest pressure (ARP).

**Results:** Followed up from 6 months to 4 years, the symptoms of fecal incontinence disappeared completely in 39 (84.78%) patients. Among them, 14 (30.43%) had complete disappearance of symptoms after 3 sessions of treatment, 25 (54.34%) patients had improved symptoms after 6 sessions of treatment, symptoms completely disappeared after 6 sessions of treatment, and 7 (15.22%) cases still suffered fecal incontinence mildly. The AMCP after three and six sessions in the poor group was significantly increased compared with that before treatment [(85.87 ± 31.75) mmHg vs. (135.33 ± 37.69) mmHg vs. (128.41 ± 33.45) mmHg, *P* < 0.05]. The ALCT and ARP showed the same trend, while the RRP after three and six sessions were not significant (*P* > 0.05). The mean (±SD) score of the RBFS increased from 9 to 17.40 ± 0.84 in the fair group, while it increased from 7.22 ± 0.76 to 16.58 ± 1.66 in the poor group after six sessions (*P* < 0.05).

**Conclusion:** Biofeedback therapy is a safe and effective treatment of fecal incontinence after the Soave procedure of children for Hirschsprung's disease. It is beneficial to design the individualized treatment programs for the children with varying degrees of fecal incontinence.

## Introduction

Hirschsprung's disease, or congenital aganglionic megacolon, is characterized by varying extent of contiguous aganglionosis extending from the anorectum proximally, which is caused by the failed migration of colonic ganglion cells during gestation. Intestinal obstruction is a typical symptom, and pull-through surgery is a typical method for treating HD in children. The Soave procedure is one of the main procedures for the treatment of HD. The outcomes of numerous reports indicate that impaired bowel function is common after surgical treatment, such as fecal incontinence and constipation (1), although surgical techniques have continuously improved in recent years (2–4). Fecal incontinence is the inability to control the passage of fecal contents through the anus and has enormous consequences on a patient's psychological, emotional, and social life (5, 6). The reported incidence of fecal incontinence after operative management of HD is broad because of varying definitions and methods of assessment (7, 8). Though BFT has been increasingly used in many clinical signs and symptoms, there is little report about the management of the fecal incontinence of HD after the Soave procedure. The main objective of this study is to evaluate the efficacy of BFT for the treatment of postoperative fecal incontinence of children for HD.

## Materials and Methods

### Patients

**Figure 1:**
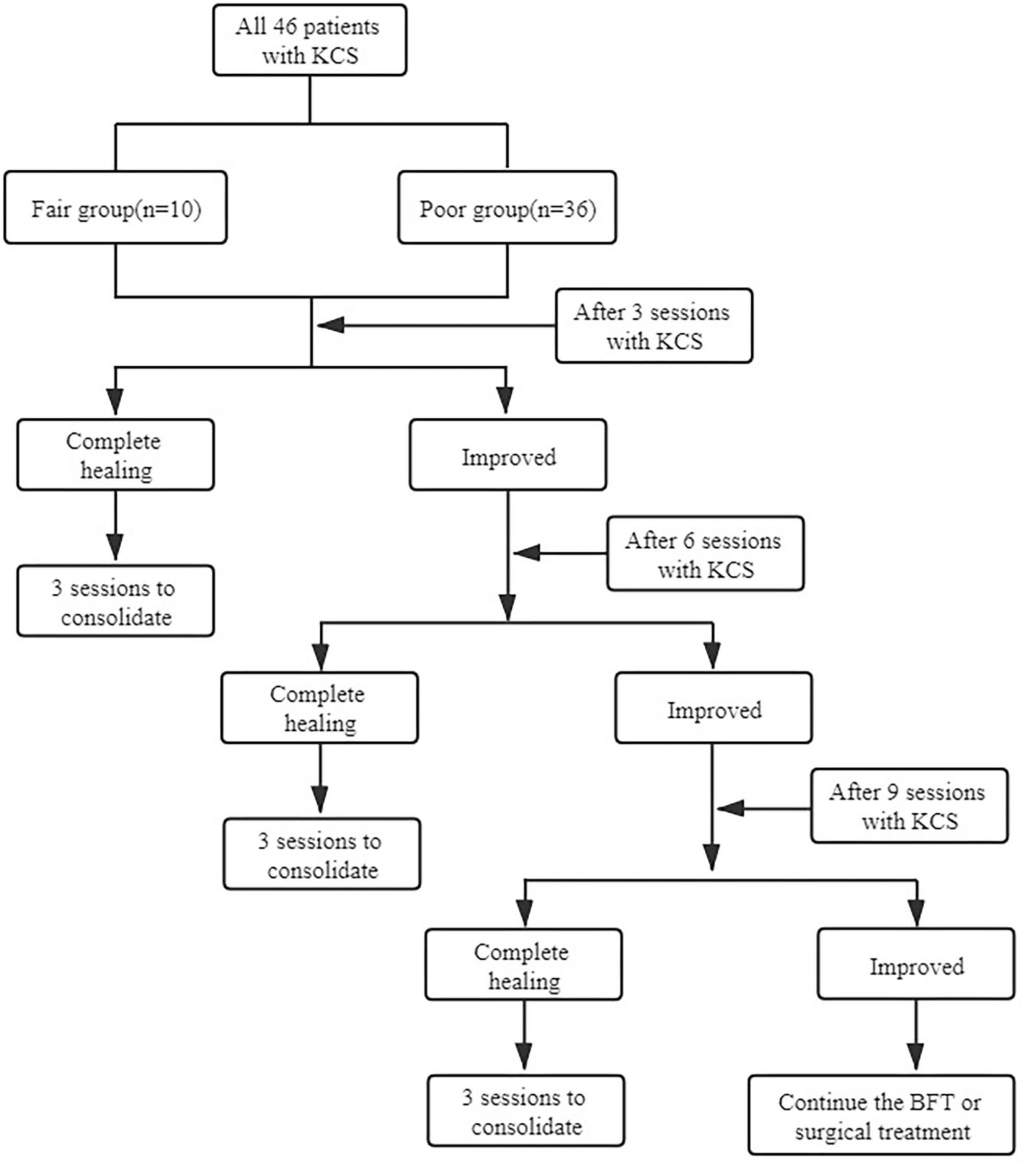
The procedures to decide the timing to terminate treatment based on clinical symptoms.

We retrospectively analyzed postoperative fecal incontinence in 46 children who received the Soave procedure for HD and who received BFT at our institution from March 2016 to February 2020, which included 38 males and 8 females (mean age 8.1 years, from 3.7 to 14 years). Exclusion criteria included children undergoing other procedures including Swenson, Duhamel, and Rehbein, combined with other digestive tract diseases, or neurogenic fecal incontinence. Anorectal manometry is a valuable diagnostic tool. The clinical outcome was evaluated by the RBFS (Table 1) (9), which considers seven basic parameters: ability to hold back defecation, feels/reports the urge to defecate, frequency of defecation, soiling, accidents, constipation, and social problems. And patients were scored according to the sum total as excellent (18–20 points, 0 case), good (11–16 points, 0 case), fair (9–11 points, 9 cases), or poor (6–9 points, 37 cases). Defecation questionnaires and anorectal manometry were completed pretreatment and after three, six, or nine sessions, and the treatment options were decided based on whether the symptoms have improved completely. The procedures are shown in Figure 1.

**Table 1:** Rintala bowel function score.

	**Score**
**Ability to hold back defecation**	
Always	3
Problems <1/week	2
Weekly problems	1
No voluntary control	0
**Feels/reports the urge to defecate**	
Always	3
Most of the time	2
Uncertain	1
Absent	0
**Frequency of defecation**	
Every other day to twice a day	2
More often	1
Less often	1
**Soiling**	
Never	3
Staining less than 1/week, no change of underwear required	2
Frequent staining, change of underwear often required	1
Daily soiling, requires protective aids	0
**Accidents**	
Never	3
Fewer than 1/week	2
Weekly accidents; often requires protective aids	1
Daily, requires protective aids during day and night	0
**Constipation**	
No constipation	3
Manageable with diet	2
Manageable with laxatives	1
Manageable with enemas	0
**Social problems**	
No social problems	3
Sometimes (foul odors)	2
Problems causing restrictions in social life	1
Severe social and/or psychic problems	0

### Biofeedback Therapy Protocol

**Figure 2:**
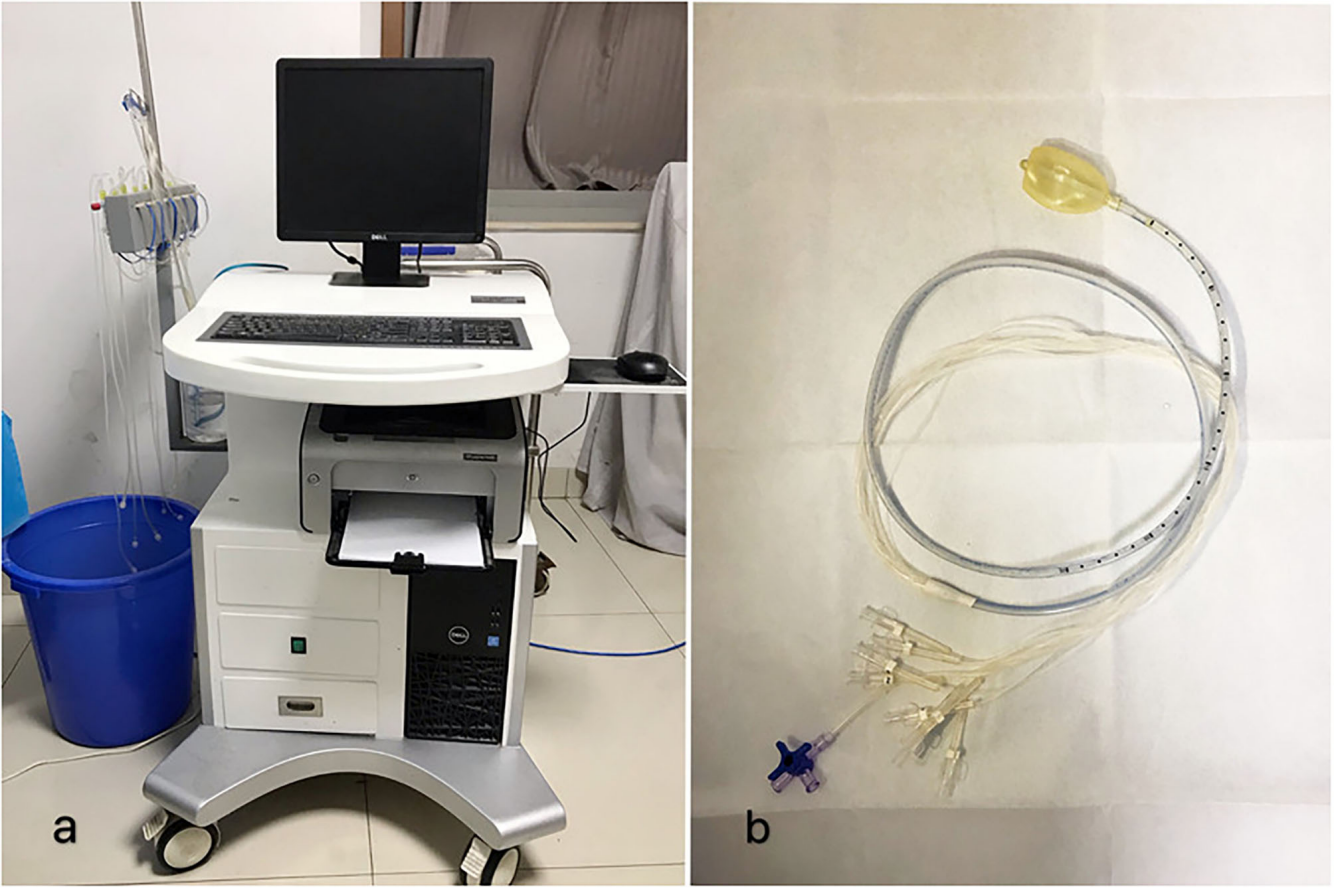
**(a)** The biofeedback instrument (XDJ-S8G, KaiLi, HeFei, AnHui, China). **(b)** The BFT catheter.

Informed consent was obtained from the parents, and this project was approved by the institutional review board of the hospital. Anal sphincter contraction training was performed using BFT by employing visual and verbal feedback techniques and by using the biofeedback instrument [XDJ-S8G, KaiLi, HeFei, AnHui, China (Figure 2a)]. Children were in the supine position with a catheter (Figure 2b) in the anus. Anal sphincter contraction or relaxation signals could be transformed into signals and displayed on the screen. Children could watch the signal on the screen and try to adjust the strength of abdomen pressure and anal sphincter contraction or relaxation. BFT included contraction training to improve the strength and persistence of the anal sphincter contraction, relaxation training to improve the relaxation of the anal sphincter, and coordinate training to remodel the anus defecation movement and improve the defecation movement coordination, for 10 days per session in the hospital outpatient department, 1 time each day, and 20 min each time. The time in between different biofeedback sessions is 1 week. The coordinate training was the hardest part of which the goal was to produce an adequate abdominal push effort, as reflected by an increase in intra-abdominal/intrarectal pressure, which was synchronized with anal relaxation, as reflected by a decrease in the anal sphincter pressure (10). Meanwhile, patients were provided advice regarding bowel habits, exercise, laxatives, dietary fiber, and fluid intake. The therapist taught subjects how to improve their push effort by using postural and diaphragmatic breathing techniques (11). All patients completed the training regimen and were followed up for 6 months to 4 years.

### Outcome Measurement

Anorectal manometry was used to evaluate the degree of fecal incontinence in all children before and after each course of treatment. Primary outcome measures were AMCP, ALCT, RRP, and ARP. The clinical outcome was evaluated by the RBFS (1, 12, 13).

### Statistical Analysis

The data were analyzed using SPSS software for Windows (version 22.0). Analysis of variance was applied to evaluate the changes between baseline and post-treatment values within the BFT. Data were presented as the mean ± standard deviation (SD). Significant differences were considered when *P* < 0.05.

## Results

A total of 46 patients (38 males and 8 females; mean age 8.1 years, from 3.7 to 14 years; mean duration of postoperative fecal incontinence, 24.0 months) were included in this analysis. All of the patients completed the study, and the severity of the phenotype showed rectosigmoid segment (14 patients), long segment (27 patients), and total colonic (5 patients) aganglionosis. All of the children went through the Soave procedure at different ages (3 cases in the neonatal period, 26 cases in 3–6 months, 5 cases in 6 months to 1 year, and 12 cases after 1 year). The demographic characteristics and the clinical spectrum of the patients are shown in Table 2.

**Table 2:** Demographic characteristics and clinical spectrum of patients.

**Characteristics**		**Number (%)**
Age	Mean Range	8.1 3.7–14
Gender	Male Female	38 (82.6) 8 (13.3)
Severity of the phenotype	Rectosigmoid segment Long segment Total colonic Neonatal period	14 (30.4) 27 (58.7) 5 (10.9) 3 (6.5)
Age at surgery	3–6 months 6 months to 1 year After 1 year	26 (56.5) 5 (10.9) 12 (26.1)
Classification	Excellent Good Fair Poor	0 (0) 0 (0) 9 (19.6) 37 (80.4)

All 46 patients were classified in the analysis as belonging to either the excellent (0 cases), good (0 cases), fair (9 cases), or poor (37 cases) group according to the RBFS. The AMCP after three and six sessions in the poor group was significantly increased compared with that before treatment [(66.10 ± 39.17) vs. (110.65 ± 26.34) vs. (94.41 ± 31.02) mmHg, *P* < 0.05], while it showed [(85.87 ± 31.75) vs. (135.33 ± 37.69) vs. (128.41 ± 33.45) mmHg, *P* < 0.05] in the fair group. The ALCT after three and six sessions in the poor group was significantly increased compared with that before treatment [(20.99 ± 12.47) vs. (38.10 ± 8.85) vs. (47.93 ± 26.95) s, *P* < 0.05], while it had the same tendency in the fair group [(27.34 ± 12.21) vs. (45.45 ± 14.76) vs. (65.55 ± 30.13) s, *P* < 0.05]. The ARP after three and six sessions in the poor group was significantly increased compared with that before treatment [(41.41 ± 23.95) vs. (67.51 ± 26.41) vs. (58.61 ± 21.97) mmHg, *P* < 0.05], while it showed [(49.17 ± 31.88) vs. (53.03 ± 23.41) vs. (62.88 ± 29.61) mmHg, *P* < 0.05] in the fair group. There was no significant change in RRP after three or six sessions in both the fair [(6.16 ± 5.67) vs. (6.97 ± 6.21) vs. (6.48 ± 4.59) mmHg, *P* > 0.05] and poor groups [(6.54 ± 5.91) vs. (5.76 ± 4.70) vs. (6.81 ± 3.69) mmHg, *P* > 0.05]. The effect of biofeedback on the anorectal manometry parameters of the patients is shown in Table 3.

**Table 3:** Effect of biofeedback on anorectal manometry parameters of patients.

	**Fair**				**Poor**			
	**Pre-biofeedback**	**After 3 sessions**	**After 6 sessions**		**Pre-biofeedback**	**After 3 sessions**	**After 6 sessions**	
AMCP (mmHg)	85.87 ± 31.75	135.33 ± 37.69	128.41 ± 33.45	*P <* 0.05	66.10 ± 39.17	110.65 ± 26.34	94.41 ± 31.02	*P <* 0.05
ALCT(s)	27.34 ± 12.21	45.45 ± 14.76	65.55 ± 30.13	*P <* 0.05	20.99 ± 12.47	38.10 ± 8.85	47.93 ± 26.95	*P <* 0.05
ARP (mmHg)	49.17 ± 31.88	53.03 ± 23.41	62.88 ± 29.61	*P <* 0.05	41.41 ± 23.95	67.51 ± 26.41	58.61 ± 21.97	*P <* 0.05
RRP(mmHg)	6.54 ± 5.91	5.76 ± 4.70	6.81 ± 3.69	*P* > 0.05	6.16 ± 5.67	6.97 ± 6.21	6.48 ± 4.59	*P* > 0.05

Different results were presented with regard to AMCP and the cure rate before and after biofeedback therapy for varying degrees of fecal incontinence (Table 4). After biofeedback therapy, the dyssynergic pattern of defecation was entirely corrected in 39 patients (84.8%), among which 14 cases (30.4%) were entirely corrected after three sessions of treatment, and 25 cases (54.3%) were entirely corrected after six sessions of treatment. Seven patients (15.2%) still had fecal incontinence after six sessions of treatment (Table 5). The seven patients had a poor prognosis after six sessions, the severity of the phenotype of which showed rectosigmoid segment (two patients), long segment (two patients), and total colonic (three patients). The ages at surgery ranged from 3 months to 1 year (six cases in 3–6 months, one case in 1 year). The ages at biofeedback treatment ranged from 3 to 14 years (Table 6). The mean (± SD) score of the RBFS group increased from 9 to 17.40 ± 0.84 in the fair group, while it increased from 7.22 ± 0.76 to 16.58 ± 1.66 in the poor group at the end of treatment (*P* < 0.05).

**Table 4:** The AMCP before and after biofeedback therapy for varying degrees of fecal incontinence.

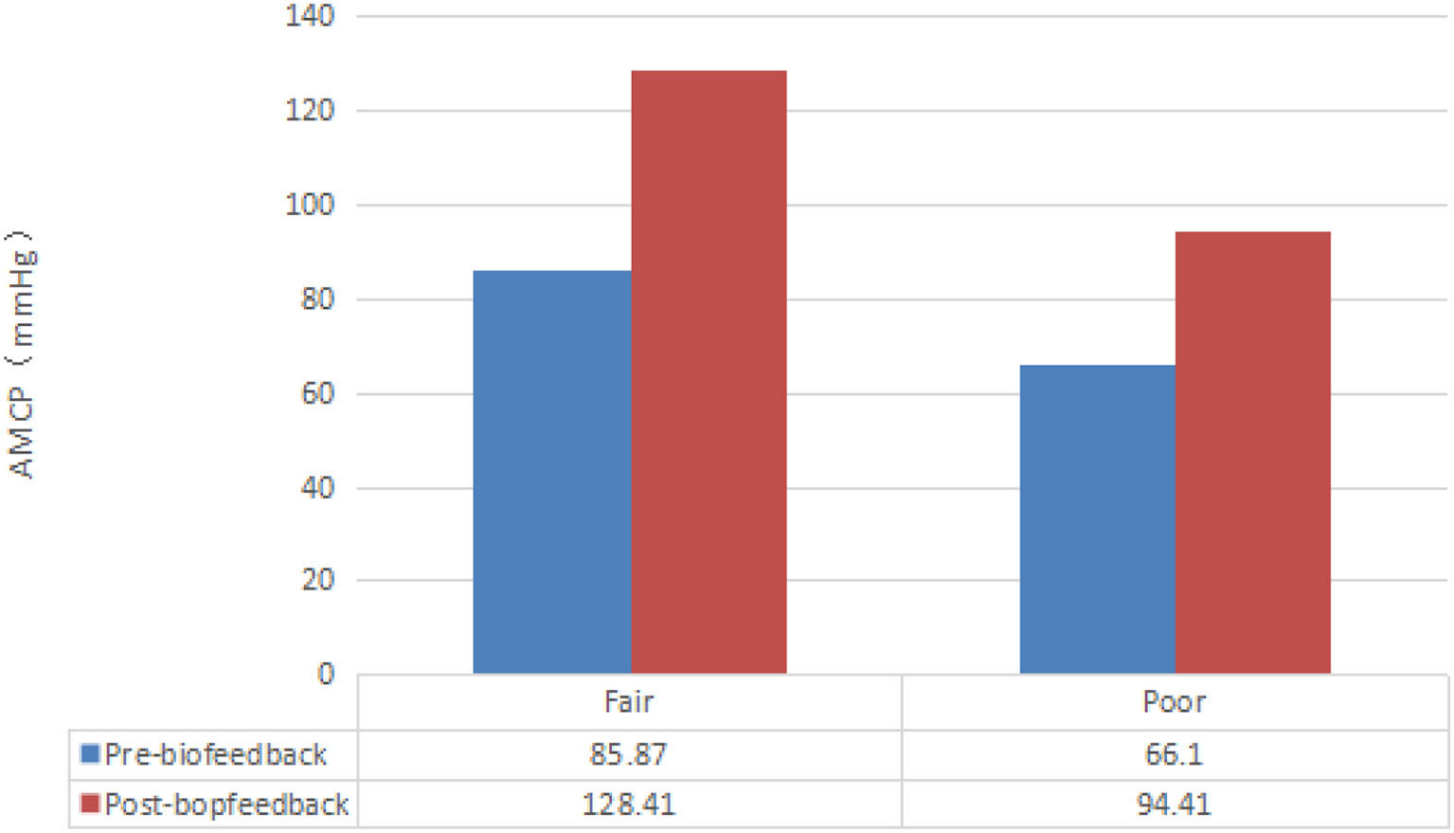

**Table 5:** The cure rate of different degree of fecal incontinence.

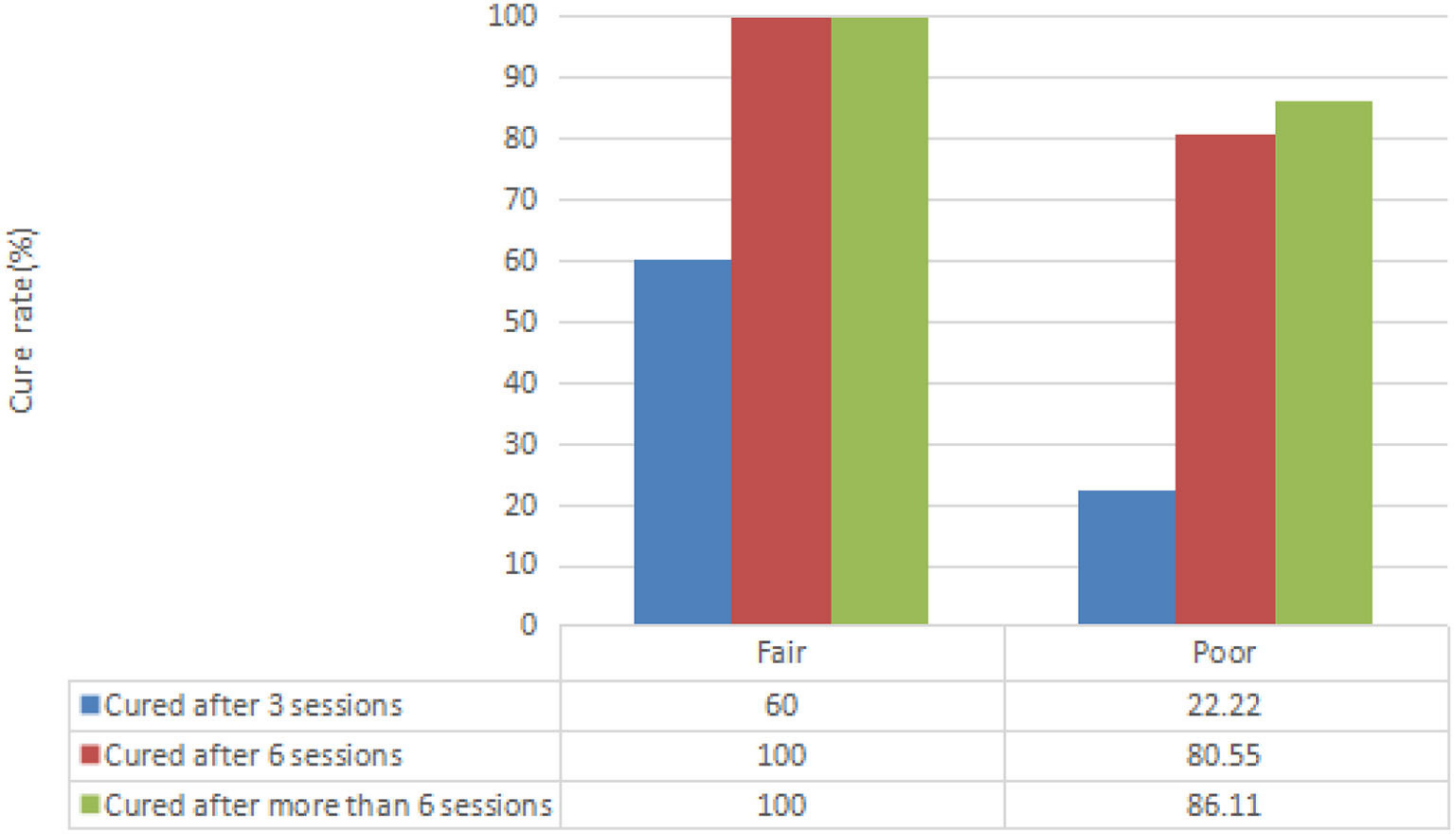

**Table 6:** Demographic characteristics and clinical spectrum of the seven patients who had a poor prognosis after six sessions.

	**Gender**	**Severity of the phenotype**	**Age at surgery**	**Age at BFT**	**RBFS before BFT**	**RBFS after BFT**
1	Male	Long segment	4 months	3 years	6	14
2	Male	Total colonic	6 months	11 years	7	11
3	Male	Rectosigmoid segment	1 year	4 years	7	13
4	Male	Total colonic	3 months	14 years	6	13
5	Male	Long segment	3 months	5 years	7	14
6	Female	Total colonic	3 months	9 years	6	15
7	Male	Rectosigmoid segment	6 months	7 years	6	16

## Discussion

Hirschsprung's disease is a common digestive tract malformation in children, which affects about 1 in 5,000 people worldwide, with the highest incidence in Asia. Operation appears to be the most effective way to manage it. Several operating procedures have been reported for treating HD (14), and the Soave procedure is one of the classic surgical methods for HD (15). While most of the patients will be able to regain normal bowel function 3 months later after the operation, fecal incontinence is common after surgical treatment, which can cause significant distress on the quality of life and social of the patients. Biofeedback is a conditioning treatment where information about a physiologic process (contraction and relaxation of a muscle) is converted to a simple visual or auditory signal to enable the patient to learn to control the disordered function, which has been increasingly used over the last decades in many clinical signs and symptoms including rehabilitation (16), tension-type headache (17), psychiatric disorders (18), and other areas like sport (19). Engel et al. first pointed out in 1974 that biofeedback training could be used to treat fecal incontinence due to sphincter damage (20, 21).

Peña and Levitt proposed that the mechanisms needed to maintain continence are intact sensation, voluntary sphincter control, and appropriate colonic motility. Loss of any of these three mechanisms can alter the patient's ability to have voluntary bowel movements (22). Xiaobing Sun et al. believed that internal sphincter damage is one important cause for fecal incontinence after the Soave procedure. The damage of the internal anal sphincter could be caused by a lower level of dissection, vigorous anal dilation, and excessive anal canal traction during operation (23).

In recent years, it is widely believed that biofeedback therapy can enhance the contraction response and strength of the anal sphincter so as to improve the ability of defecation control (24). The efficacy could be measured by anorectal manometry. Our experience in this study shows that the ARP before treatment was at a lower level compared to baseline, which reflects the impaired function of the anal sphincter, and may be the main cause of postoperative fecal incontinence, consistent with relevant literature reports. It also shows the positive effects of biofeedback on most physiological parameters, including AMCP, ALCT, and ARP, in children with postoperative fecal incontinence, and patients with severe clinical symptoms need more courses of treatment to recovery. As it turns out, after six sessions, AMCP and ALCT were more than double compared with that before treatment, while ARP was also increased obviously. These advances could provide adequate relief in fecal incontinence, and satisfaction with bowel movement in patients after treatment was significantly higher than before treatment. The clinical outcome was evaluated by the RBFS, of which an advantage is that it quantifies clinical outcomes, allowing us to more intuitively compare outcomes before and after treatment. The above two reflect subjective and objective indicators, respectively.

We observed a curious phenomenon in this research in which the AMCP had a trend of increasing significantly and then decreasing slightly. This brings us to an important point: the AMCP does not continue to increase with the number of sessions. We surmise that with the increase in the treatment course, patients may feel constant tiredness, so that the AMCP may decrease slightly after six sessions; but it was not statistically significant and therefore has little effect on prognosis.

It was shown that it requires different sessions of treatment according to different degrees of fecal incontinence. This paper will be improved by a data analysis of predictive factors to the biofeedback that provided the worse results. Seven patients had a poor prognosis after six sessions, the severity of the phenotype of which showed rectosigmoid segment (two patients), long segment (two patients), and total colonic (three patients). We observed that the admission to surgery or BFT of the two patients who showed rectosigmoid segment was delayed. Therefore, major factors leading to a poor prognosis may be associated with the aganglionic length, the age at surgery, as well as the age at biofeedback treatment. Biofeedback therapy requires children to understand the treatment process, cooperate actively, and insist on active exercise. Therefore, it is also important to select appropriate ages for treatment. Some children have poor autonomy and poor self-consciousness in training, which also have an impact on the treatment effect. Therefore, we believe that BFT should adhere to long-term exercise.

## Conclusion

Postoperative fecal incontinence of children for HD can be a heavy burden for many children and their families. At our institution, BFT appears to result in a significant improvement in AMCP, ALCT, ARP, and the RBFS, so we believe that biofeedback therapy is a safe and effective treatment. It is beneficial to design the individualized treatment programs for the children with varying degrees of fecal incontinence. BFT does not have a uniform standard protocol for fecal incontinence of different types; we attempt to create a standardized protocol to reduce pain and improve the quality of life of children who suffer from fecal incontinence. Furthermore, the lack of long-term reassessment of results is a major limitation of this study, so long-term follow-up will continue in order to make the results reproducible and reliable.

## Data Availability Statement

The raw data supporting the conclusions of this article will be made available by the authors, without undue reservation.

## Ethics Statement

The studies involving human participants were reviewed and approved by Ethics Committee Office of the First Affiliated Hospital of Zhengzhou University. Written informed consent to participate in this study was provided by the participants' legal guardian/next of kin.

## Author Contributions

HY designed the experiment and modified the paper. YY and MX performed the experiment. MX and GW processed the data. YY wrote the paper. All authors contributed to the article and approved the submitted version.

## Conflict of Interest

The authors declare that the research was conducted in the absence of any commercial or financial relationships that could be construed as a potential conflict of interest.

## Publisher's Note

All claims expressed in this article are solely those of the authors and do not necessarily represent those of their affiliated organizations, or those of the publisher, the editors and the reviewers. Any product that may be evaluated in this article, or claim that may be made by its manufacturer, is not guaranteed or endorsed by the publisher.
